# Neutrophil-Mediated Endogenous Analgesia Contributes to Sex Differences in Oral Cancer Pain

**DOI:** 10.3389/fnint.2018.00052

**Published:** 2018-10-22

**Authors:** Nicole N. Scheff, Aditi Bhattacharya, Edward Dowse, Richard X. Dang, John C. Dolan, Susanna Wang, Hyesung Kim, Donna G. Albertson, Brian L. Schmidt

**Affiliations:** ^1^Bluestone Center for Clinical Research, New York University, New York, NY, United States; ^2^College of Dentistry, New York University, New York, NY, United States

**Keywords:** sex differences, neutrophils, endogenous analgesia, squamous cell carcinoma, pain, opioids, orofacial, cancer

## Abstract

The incidence of oral cancer in the United States is increasing, especially in young people and women. Patients with oral cancer report severe functional pain. Using a patient cohort accrued through the New York University Oral Cancer Center and immune-competent mouse models, we identify a sex difference in the prevalence and severity of oral cancer pain. A neutrophil-mediated endogenous analgesic mechanism is present in male mice with oral cancer. Local naloxone treatment potentiates cancer mediator-induced orofacial nociceptive behavior in male mice only. Tongues from male mice with oral cancer have significantly more infiltrating neutrophils compared to female mice with oral cancer. Neutrophils isolated from the cancer-induced inflammatory microenvironment express beta-endorphin and met-enkephalin. Furthermore, neutrophil depletion results in nociceptive behavior in male mice. These data suggest a role for sex-specific, immune cell-mediated endogenous analgesia in the treatment of oral cancer pain.

## Introduction

Oral cancer is the sixth most common cancer worldwide (Parkin et al., 2005); more than 90% of oral cancers are squamous cell carcinoma (SCC; Pai and Westra, 2009). While the global incidence of oral cancer has been decreasing due to a reduction in smoking, the incidence of oral cancer in the USA is increasing, especially in young people and women (Hussein et al., 2017; Tota et al., 2017). Orofacial pain is the most common initial symptom that leads to the diagnosis of oral cancer in patients (Marshall and Mahanna, 1997; Lam and Schmidt, 2011) and impairs speech, eating, drinking, and interpersonal relations (Bjordal et al., 2001). Preoperative oral cancer pain also predicts poor patient outcomes (Reyes-Gibby et al., 2014). The opioid requirement for oral cancer pain is high. While there is some pain relief, opioids generate debilitating side effects including respiratory depression, nausea, constipation and sedation. The reports on the prevalence and intensity of oral cancer pain in women and men are contradictory; previous studies have reported greater pain in men (Connelly and Schmidt, 2004; Cuffari et al., 2006), equal pain between the sexes (Sato et al., 2010) and greater pain in women (Reyes-Gibby et al., 2014). The discrepancies between these reports are most likely due to small sample size and/or an incomplete phenotypic characterization of cancer pain. Thus, there is a clinical need to understand the sex difference in the experience of oral cancer pain in women and men.

Across all orofacial pain conditions, prevalence is higher in women (Dao and LeResche, 2000; Fillingim et al., 2009). This sex difference is attributed in part to gonadal hormones, which have effects throughout the peripheral and central nervous systems (CNSs), including pain and analgesic signaling (Craft et al., 2004; Craft, 2007; Fillingim et al., 2009). Hormones also influence inflammation which is involved in pain pathophysiology (Grace et al., 2014). Women show a heightened inflammatory response compared with men in response to sepsis and the proinflammatory effects of chronic autoimmune diseases (Straub, 2007). Sex differences in the role of immune cell signaling in mechanical pain hypersensitivity have been reported in the CNS (Sorge et al., 2015). Microglia, fundamental to neuropathic pain signaling in males, are not involved in female pain processing; female mice achieve similar levels of pain hypersensitivity using adaptive immune cells, likely T lymphocytes (Sorge et al., 2015). We described a role for peripheral infiltrating T cells in oral cancer pain in female mice (Scheff et al., 2017). The role of infiltrating immune cells in oral cancer pain in males, however, has not been investigated.

While the immune system is known to contribute to inflammatory pain, it can also be responsible for an endogenous opioid-based mechanism of pain control (Hua, 2016). Clinical and animal studies demonstrate that under environmental stressful stimuli immune cells can secrete opioids, which activate opioid receptors localized on peripheral sensory nerves to produce analgesia (Stein et al., 1991, 2003; Mousa et al., 2007; Hua and Cabot, 2010). Endogenous mediators that trigger opioid release within inflamed tissue are corticotropin-releasing factor (CRF) and cytokines (Stein, 1995; Stein and Yassouridis, 1997). Opioid peptides, β-endorphin and met-enkephalin are found in human synovial cells as well as in lymphocytes, macrophages and mast cells; only minor amounts of dynorphin are detectable (Stein et al., 1996). In the early stage of inflammation, neutrophils are the major opioid-containing leukocyte, whereas monocytes/macrophages and lymphocytes predominate at later stages (Rittner et al., 2001; Brack et al., 2004).

To investigate sex differences in oral cancer pain in patients with oral SCC, we utilize the University of California San Francisco Oral Cancer Pain Questionnaire (UCSFOCPQ) to quantify pain and to identify functions that generate oral cancer pain (Connelly and Schmidt, 2004). The UCSFOCPQ measures oral cancer pain and is the only validated instrument (Kolokythas et al., 2007). There is a lack of information regarding sex differences in nociceptive behavior in oral cancer pain animal models. We use preclinical immune competent models to study oral cancer pain in female and male rodents. We find that women experience greater oral cancer pain and that neutrophil-mediated endogenous analgesia contributes to suppression of pain in male mice.

## Materials and Methods

### UCSF Oral Cancer Pain Questionnaire

The UCSFOCPQ was administered to 72 oral cancer patients (35 women, 37 men; mean age = 64.5 ± 14.4 (SD) years) referred to the New York University Oral Cancer Center from 2010 to 2017. The UCSFOCPQ was administered to patients at a preoperative clinic visit before being prescribed analgesics for their oral cancer pain and before any treatment, as described previously (Kolokythas et al., 2007). Inclusion criteria for patients in the study were diagnosis of oral cancer and comprehension of the UCSFOCPQ. Exclusion criteria included a diagnosed psychiatric condition, addiction to pain medications or recreational drugs, and analgesic usage in the previous 6 months. Detailed demographic information, which included age, sex, anatomic location of the oral cancer, stage and evidence of metastasis is presented in Supplementary Table S1. The study was carried out in accordance with the recommendations of the Institutional Review Board at New York University. All subjects gave written informed consent in accordance with the Declaration of Helsinki. The validated questionnaire (Supplementary Figure S1) comprises eight questions that are answered by the patient on a visual analog scale of 0–100 mm (Kolokythas et al., 2007; Lam and Schmidt, 2011). The questions differentiate spontaneous vs. function-related pain and characterize pain quality. Questions one through six elicit patient response related to pain intensity, sharpness, and ache in the orofacial region. Question seven concerns sensitivity to touch. Question eight queries functional restriction secondary to orofacial pain. Patients were instructed to place a vertical line along the 100 mm horizontal scale to approximate their orofacial pain level (if any) for each question.

### Cell Culture and Supernatant Collection

Human oral cancer cell line, HSC-3, and non-tumorigenic keratinocyte cell line, HaCaT, were utilized to produce the acute supernatant cancer pain and control models, respectively. Cells were maintained and culture supernatant was collected as previously described (Scheff et al., 2017). All cell lines were cultured in 10 cm^2^ cell culture dishes at 37°C with 5% CO_2_ in Dulbecco’s modified Eagle’s medium (DMEM, Gibco, Waltham, MA, USA) supplemented with 10% fetal bovine serum and penicillin/streptomycin (50 U/mL). For collection of supernatant for the acute pain model, the culture medium was changed to serum-free DMEM without phenol red (3 mL total volume) when cells reached 70%–80% confluency (1.5 × 10^6^ cells) and cells were incubated for a further 48 h. Cell culture supernatant was collected, centrifuged at 300× *g* to remove cell debris, and frozen at −20°C until needed. HSC-3 and HaCaT cell culture supernatant was collected from passage 8 and 11, respectively.

### Animals

Adult (10–12 weeks, 20–30 g) female and male C57BL/6 mice (stock #000664, Jackson Labs, Bar Harbor, ME, USA) were used for all experiments. All mice were housed in a temperature-controlled room on a 12:12 h light:dark cycle (07:00–19:00 h light), with unrestricted access to food and water. All animal experiments were carried out in accordance with the recommendations of the National Institute of Health guidelines and the PHS Policy on the Humane Care and Use of Laboratory Animals. The protocol was approved by the New York University Institutional Animal Care and Use Committee.

### Orofacial Behavior

The dolognawmeter, a device and assay, was designed to quantify gnawing activity. The outcome variable (gnaw-time for the second dowel) is a validated index of orofacial nociception in mice with oral cancer (Dolan et al., 2010). Each mouse was placed into a confinement tube; forward movement in the dowel is obstructed by two dowels in series in front of the mouse. The mouse voluntarily gnaws through both dowels to escape the device. Each obstructing dowel is connected to a digital timer. The timer automatically records the duration required for the mouse to sever the dowel. To acclimatize the mice and improve consistency in gnawing behavior, all mice were trained for 5–7 sessions in the dolognawmeter. Training is accomplished by placing the mice in the device and allowing them to gnaw through the obstructing dowels in the same manner as the subsequent experimental gnawing trials. For both oral cancer pain models, a baseline gnaw-time (mean of the final three training sessions) was established for each mouse. The investigator was blinded to the treatment groups. After baseline gnaw-times were determined, treatment or drug injections were initiated and the mice underwent behavioral testing one time per week for 28 weeks. Each mouse was compared to its own baseline gnaw-time and data are presented as a percent change ± standard error of the mean.

### Oral Cancer Pain Models

#### Acute Oral Cancer Pain Model

We developed an acute oral cancer pain model by injecting cell culture supernatant into the tongue (Scheff et al., 2017). Mice received 50 μl injections (under isoflurane general anesthesia) of either HSC-3 or HaCaT cell culture supernatant over a 5 s period, into the left lateral tongue for three consecutive days. Nociceptive orofacial behavior measurements using the dolognawmeter assay and device were recorded in awake mice 1 h after the third supernatant injection. Inflammatory infiltrate was measured using flow cytometry 12 h after the third injection. Naloxone (500 μg/kg; Sigma Aldrich, St. Louis, MO, USA) was co-injected with HSC-3 cell culture supernatant for three consecutive days in between behavioral assay trials for experiments designed to inhibit endogenous opioid-mediated analgesic signaling in response to oral cancer supernatant in female and male mice. Data is displayed as three stable baseline gnaw-time measurements followed by one gnaw-time measurement following the three injections. Data is analyzed as a percent change from an average across the three baseline gnaw-times.

#### 4-Nitroquinoline-1-Oxide (4NQO)-Induced Oral Cancer Pain Model

To study the development of oral nociception with carcinogenesis, female and male mice were first trained in the dolognawmeter over 5–7 sessions (Dolan et al., 2010). Subsequently these mice were offered carcinogen 4-nitroquinoline-1-oxide (4NQO; 100 μg/mL; Sigma Aldrich, St. Louis, MO, USA) in their drinking water or the equivalent dilution of the vehicle, propylene glycol for 16 weeks (Scheff et al., 2017). The mice were then monitored weekly under light anesthesia for tumor incidence, location and size for an additional 12 weeks. Functional allodynia, resulting from 4NQO-induced carcinogenesis, was assessed using the dolognawmeter assay and device once per week for the entire duration of the model (28 weeks); gnaw-time in seconds was used as a behavioral index of functional mechanical allodynia (Figure 2A). Tongue tissue was then harvested and a 1–2 mm coronal section was dissected from the most clinically suspicious region, fixed in 10% formalin, and processed for paraffin embedding and slide preparation. Three 5 μm hematoxylin & eosin stained tongue sections separated by 100 μm were evaluated for the presence of papillary and invasive SCC and the remaining tissue was used for flow cytometry or protein quantification. Histopathologic analysis was performed by an oral and maxillofacial pathologist (AB) blinded to group identity. Only mice with histologically confirmed papillary and/or invasive lesions were included in the analysis of nociceptive behavior. Out of 55 mice, we identified six invasive lesions (female *n* = 2, male *n* = 3) and 21 papillary lesions (female *n* = 12, male *n* = 19). All invasive lesions were accompanied by a papillary component. Three male mice and two female mice had ≥2 distinct papillary lesions identified in the biopsy. We recognize that papillary and invasive cancers are histologically distinct subtypes, which may have dissimilar interactions with the tongue microenvironment impacting nociceptive behavior. However, for this initial analysis we have chosen to combine both lesion subtypes to a single group for comparison to vehicle-treated mice.

**Figure 1:**
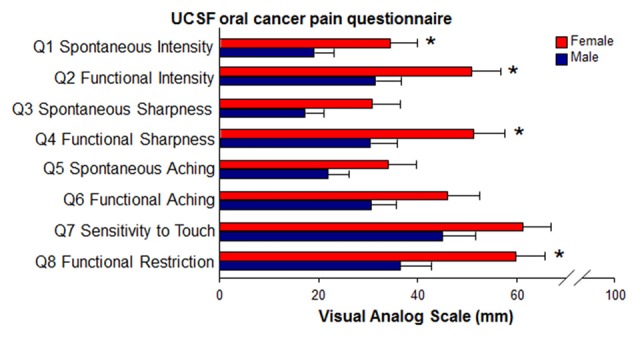
Sex differences in reported pain in oral cancer patients. Oral cancer patients (35 women, 37 men) completed the University of California SanFrancisco Oral Cancer Pain Questionnaire (UCSFOCPQ, Supplementary Figure S1). Mean visual analog scale pain scores for each question were compared between women and men. Women (red bars) experienced significantly more spontaneous and function-related pain, as well as increased functional restriction compared to men (blue bars). *P < 0.05 by Mann-Whitney *U*.

**Figure 2:**
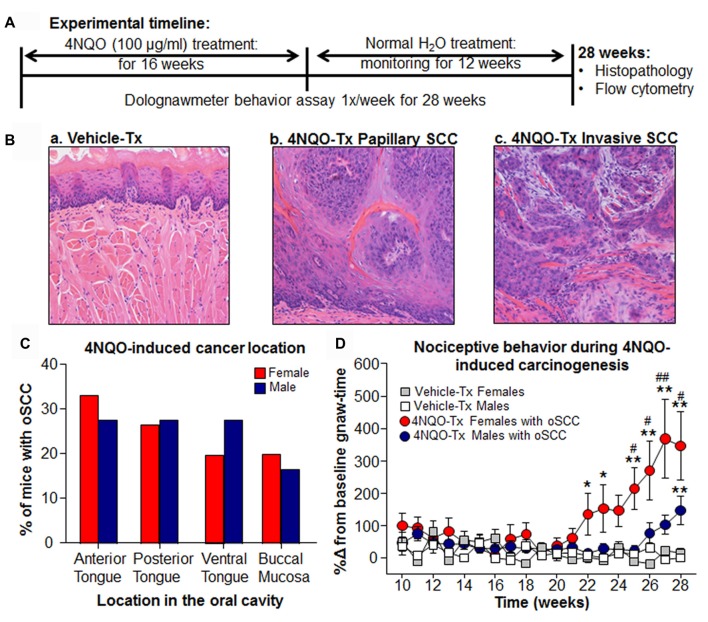
4-nitroquinoline-1-oxide (4NQO)-induced mouse model of oral squamous cell carcinoma (oSCC). **(A)** Schematic of the experimental design. Female (*N* = 50) and male (*N* = 50) mice received either 4NQO or propylene glycol (vehicle) in the drinking water *ad lib* for 16 weeks. The mice were monitored for oral lesions for an additional 12 weeks. Gnawing function was quantified weekly with a dolognawmeter. Tongue tissues were then processed for histopathology and flow cytometry. **(B)** Representative hematoxylin & eosin stained tongue sections from a vehicle-treated (tx) male mouse (**a**; 4× with 10× inset), male mouse with 4NQO-induced papillary lesion (**b**; 4× with 10× inset) on the posterior tongue, and invasive lesion (**c**; 4× with 10× inset) on the anterior tongue. **(C)** Pooled data from 15 female and 16 male mice with 4NQO-induced lesions occurring on posterior tongue, anterior tongue, ventral tongue and buccal mucosa. Data represented as a percent of total mice by sex. **(D)** Functional allodynia was analyzed as mean gnaw-time across mice with 4NQO-induced oSCC or vehicle-treatment over time (weeks). Pooled data from female (red circles) and male (blue circles) mice with 4NQO-induced oral SCC (4NQO-Tx) and vehicle-treated mice (Vehicle-Tx, squares) over time analyzed by Three-way analysis of variance (ANOVA). Holm-Sidak *post hoc* comparisons for Sex by Time, ^#^*P* < 0.05, ^##^*P* < 0.01; Time by Treatment, *P < 0.05, **P < 0.01.

### Tongue Immune Cell Quantification and Sorting

#### Tongue Tissue Dissociation

Tongue tissue was dissected into cold DMEM containing antibiotics (penicillin/streptomycin, 10 U/mL) and 20 mM HEPES and was dissociated as previously described (Scheff et al., 2017). Tongue tissue was dissected and minced in DMEM with antibiotics, collagenase-H (0.5 mg/mL; Sigma Aldrich, 34 Units/mg), DNase (0.5 mg/mL) and 20 mM HEPES, and then incubated at 37°C for 1 h. The tissue was then mechanically dissociated using a fire-polished pipette, washed twice with fresh DMEM containing antibiotics and HEPES, and resuspended in Ca^2+^/Mg^2+^ free phosphate buffered saline (Sigma Aldrich) containing 3% fetal bovine serum, 1 mM EDTA, and 0.02% sodium azide and filtered through a 40 μm cell strainer (Falcon brand, Fisher Scientific, Waltham, MA, USA).

#### Flow Cytometry

Flow cytometry was used to characterize immune cell types in the tongue tissue from female and male mice with 4NQO-induced oral SCC compared to tongue tissue in vehicle-treated female and male mice using the antibody panel and flow cytometry gating strategy as previously defined (Scheff et al., 2017). Within CD45^+^ hematopoietic cells, six subpopulations were detected and quantified using antibodies specific to receptors expressed on each cell type. Single-cell suspensions were prepared as described above and samples were incubated in rat anti-mouse purified CD16/CD32 to block unspecific FC receptor binding. CD45 monoclonal antibody (mAb) conjugated with V450 dye (1:400; BD Biosciences Franklin Lakes, NJ, USA) was used to label all hematopoietic cells. To differentiate leukocyte subpopulations, we stained cell suspensions with fluorescently conjugated rat anti-mouse mAbs recognizing neutrophils (Ly6G, Cat# 561105, 1:500), monocytes/macrophages (CD11b, Cat# 561690, 1:1,000), dendritic cells (CD11c, Cat# 561044, 1:250), T lymphocytes (CD3, Cat# 561824, 1:250), natural killer cells (NK1.1, Cat# 561111, 1:300), or B cells (CD45R/B220, Cat# 561877, 1:500; all BD Biosciences). The specificity of the staining was verified by incubation of cell suspensions with appropriate isotype-matched control antibodies. The gating strategy for isolation of these populations was to first exclude dead cells in the population using propidium iodide (PI; Molecular Probes, Eugene, OR, USA). Of the recovered live cells, CD45^+^ immune cells were selected and then sorted into CD3^+^ T cells and CD3^−^ leukocytes; the latter were further sorted into CD45R^+^ B-cells or NK1.1^+^ natural killer cells. The remaining CD3^−^CD45R^−^NK1.1^−^ cells were then sorted into CD11b^+^ monocyte/macrophages/neutrophils and CD11c^+^ dendritic cells. CD11b^+^/c^−^ immune cells were further sorted into CD11b^+^/Ly6G^+^ and Ly6G^−^ to isolate monocyte/macrophages and neutrophils respectively. Viability was uniformly 70%–85%, as determined by PI staining. An average of 8.11 × 10^5^ ± 4.1 × 10^4^ live cells were recovered from each naïve tongue. Spleen cells were used for compensation controls. Data were acquired using a Fluorescence-activated cell sorting (FACS) Calibur (BD Biosciences) and analyzed using FlowJo software (Tree Star, San Carlos, CA, USA).

#### Fluorescence-Activated Cell Sorting (FACS)

FACS was used to collect myeloid and lymphoid populations of immune cells from dissociated tongue tissue treated with HSC-3 cell culture supernatant. Tongue tissue was dissected and dissociated in a manner similar to that used for flow cytometry. To isolate subpopulations, cells were stained with fluorescently conjugated rat anti-mouse mAbs: CD450 (1:400), CD11b (1:1,000), and Ly6G (1:500). PI was used to exclude dead cells. Neutrophils were defined as CD45^+^CD11b^+^Ly6G^+^. Post-sort purity was >97%. FACS was performed on a 3 laser, 10 detector FACSAria cell sorter (BD Biosciences). Samples were sorted into phosphate buffered saline containing RNAse inhibitor, pelleted (300× g), and snap frozen in liquid nitrogen for further processing.

#### Antibody-Mediated Neutrophil Depletion

In the acute oral cancer pain model, anti-mouse Ly6G mAb (αLy6G, clone 1A8; BioXCell, West Lebanon, NH, USA) was used to specifically deplete neutrophils (Daley et al., 2008) *in vivo*. Female and male mice were trained in the dolognawmeter and then administered a single i.p. injection of αLy6G (1 mg/mouse) or isotype control IgG2A (clone 2A3) 12 h prior to three consecutive injections of HSC-3 or HaCaT cell culture supernatant into the lateral tongue. Dolognawmeter behavior measurements were recorded in awake mice 1 h after the third supernatant injection. Inflammatory infiltrate was measured using flow cytometry 12 h after the third injection.

To deplete neutrophils during 4NQO-induced carcinogenesis, male mice were trained in the dolognawmeter and then administered 4NQO treatment (100 μg/mL) or the equivalent dilution of propylene glycol in drinking water for 16 weeks. The mice were then monitored for an additional 9 weeks. Starting at week 22, mice received an i.p. injection of αLy6G (1 mg/mouse) or isotype control IgG2A every 72 h for 2.5 weeks. Orofacial behavior was measured 24 h after each injection. At week 25, the tongues were harvested for flow cytometry analysis to verify the loss of neutrophils and measure the impact of neutrophil depletion on other infiltrating immune cell subtypes.

### Electrophysiology

#### Retrograde Tracer Labeling

At least 10 days prior to tissue harvest, the retrograde tracer 1,1′-dioctadecyl-3,3,3′,3′-tetramethy lindocarbocyanine perchlorate (DiI, Invitrogen, Carlsbad, CA, USA) was injected peripherally into the anterior lateral tongue to retrograde label tongue afferents. For the 4NQO carcinogenesis model, mice were injected with DiI 10 days prior to carcinogen treatment. The tracer was dissolved at 170 mg/mL in dimethylsufoxide (DMSO), diluted 1:10 in 0.9% sterile saline, and injected bilaterally using a 30 g needle for a total volume of 5–7 μL per tongue under isoflurane (Abbott Laboratories, North Chicago, IL, USA) anesthesia.

#### Tongue Primary Afferent Primary Culture

Adult mice were anesthetized with isoflurane and transcardially perfused with cold Ca^2+^/Mg^2+^-free Hank’s balanced salt solution (Invitrogen). Bilateral trigeminal ganglia (TG) were dissected into cold Hank’s balanced salt solution and dissociated as previously described (Scheff et al., 2017). Cells were plated in Dulbecco’s Modified Eagle Medium: nutrient Mixture F12 (DMEM/F12, Gibco) containing 5% fetal bovine serum and antibiotics (penicillin/streptomycin, 50 U/mL). Coverslips were flooded 2 h later with Leibovitz’s L-15 media (Gibco) containing 10% FBS, 5 mM HEPES and 5 mM glucose, and used at room temperature. Experiments were performed within 8 h of tissue harvest.

#### Current Clamp Physiology

Whole cell patch clamp recording was used to assess changes in the excitability of cultured retrograde labeled TG neurons from naïve, vehicle-treated mice and mice with 4NQO-induced oral SCC. Borosilicate glass electrodes were filled with 110 mM K-methanesulfonate, 30 mM KCl, 5 mM NaCl, 1 mM CaCl_2_, 2 mM MgCl_2_, 10 mM HEPES, 11 mM EGTA, 2 mM Mg-ATP, 1 mM Li-GTP, pH 7.2 (adjusted with Tris-base), 310 mOsm (adjusted with sucrose). Neurons were continuously superfused with a bath solution that contained 3 mM KCl, 130 mM NaCl, 2.5 mM CaCl_2_, 0.6 mM MgCl_2_, 10 mM HEPES, 10 mM glucose, pH 7.4 (adjusted with Tris-base), 325 mOsm (adjusted with sucrose). Cell culture supernatants were applied with a computer-controlled perfusion fast-step system (switching time <20 ms; Warner Instrument Co, Hamden, CT, USA, Model SF-77B). Oral cancer-induced changes in excitability were assessed in current-clamp mode with four measures: spontaneous activity, action potential (AP) threshold, rheobase, and accommodation. Spontaneous activity was assessed at resting membrane potential (Vm) for 30 s at baseline and up to 90 s after the application of culture supernatant. The second two measures were determined with a 750 ms depolarizing square-pulse current injection. AP threshold was defined as the greatest depolarization reached before spike generation in response to depolarizing current injections. Rheobase was defined as the smallest amount of current needed to evoke a single AP. Because rheobase is positively correlated with cell size, values were normalized with respect to membrane capacitance to facilitate comparisons between neurons. Passive properties assessed included Vm, capacitance and input resistance (R_in_). R_in_ was measured with five 750 ms hyperpolarizing current injections (2–5 pA) from Vm. Active electrophysiological properties were assessed with an AP evoked by a 4 ms depolarizing current pulse. These properties included: AP duration at 0 mV, magnitude of AP overshoot, magnitude of the after-hyperpolarization (AHP), and AHP decay (τ AHP). The magnitude of the overshoot was measured from 0 mV. The magnitude of the AHP was measured from the Vm. Decay of the AHP was estimated by fitting the decay phase of the AHP with a single exponential function. Neurons with a cell body diameter greater than 10 μm and an inflection in the falling phase of the AP were included in the study. For each afferent neuron isolated for study, a continuous recording was obtained for 60 s without the delivery of external stimulus. If spontaneous discharge persisted during this period, the neuron was classified as spontaneously active and disregarded.

### Enzyme-Linked Immunosorbent Assay

The granulocyte-macrophage-colony stimulating factor (GM-CSF) protein concentration in tongue tissue from female and male mice with 4NQO-induced oral SCC compared to vehicle-treated female and male mice by ELISA (MyBioSource, Inc; San Diego, CA, USA). Frozen tissue (20–40 mg) was homogenized in the T-PER Reagent (Pierce Biotechnology, Inc., Rockford, IL, USA) and agitated for an additional 2 h at 4°C. Lysates were centrifuged at 13,000 rpm for 5 min. Cell culture supernatants were removed, aliquoted and protein concentrations were determined using a Bradford Assay (Bio-Rad Laboratories, Inc., Hercules, CA, USA). ELISA was run per the manufacturer’s instructions. The optical density of the standards and samples was read at 450 nm using a Model 680 Microplate Reader (Bio-Rad Laboratories, Inc., Hercules, CA, USA).

### Polymerase Chain Reaction (PCR)

FAC sorted immune cell populations were resuspended in RNA lysis buffer and total RNA isolation of each sample was conducted with a Zymo Research Quick-RNA MicroPrep Kit (Zymo Research, Irvine, CA, USA). The RNA (concentration ≥5 ng/μL as determined by fluorometry) was reverse transcribed into cDNA with a High Capacity cDNA Reverse Transcription Kit (Applied Biosystems Inc. Carlsbad, CA, USA) according to the manufacturer’s instructions. SYBR green-based real-time quantitative polymerase chain reaction (PCR) was used to assess relative expression of opioid precursors in FAC sorted neutrophils on a real-time thermal cycler (Agilent Technologies, Santa Clara, CA, USA) using a 40-cycle protocol with a denaturation step at 95°C for 15 s and annealing step at 60°C for 60 s. All samples were run in triplicate in a final volume of 20 μL that includes 2 μL of cDNA (2.5 ng/μL) and 10 μM of each gene specific primer. The melting curve of all PCR products produced a single peak and the gene primer efficiencies were calculated according to the equation *E* = 10^[−1/slope]^. The primer pairs for gene expression assays were purchased from Integrated DNA Technologies (IDT, Coralville, IA, USA): *Pomc* (Forward-TAGACGTCCAAACCCTCGTT; Reverse-AGCGAGAGGTCGAGTTTGC), *Penk* (Forward-CAGCCAGGACTGCGCTAAAT; Reverse-GAAGCCTCCGTACCGTTTCAT), *Pdyn* (Forward-GCCCTCTAATGTTATGGCGGA; Reverse-TCCTTCAGGACGGGTTCCAA). The housekeeping gene *Actb* (Forward-AGTGTGACGTTGACATCCGT; Reverse-GTAACAGTCCGCCTAGAAGCA) was used as the internal control gene. Relative quantification analysis of gene expression data from male mice was calculated using the pfaffl method (Pfaffl, 2001) and normalized relative to the average expression ratio in female mice.

### Statistical Analysis

The Mann-Whitney *U* test was used to evaluate sex differences in responses to the UCSFOCPQ. Unpaired *t*-test and analysis of variance (ANOVA) were employed to evaluate the difference between groups regarding sex and treatment in animal studies. Three-way ANOVA repeated measure was used to determine the difference between groups when considering sex and treatment over time in animal studies. To adjust for multiple comparisons, the *post hoc* Holm-Sidak test statistic was employed. Statistical significance was set at *p* < 0.05. Pearson correlation was used to measure the linear relationship between two variables (i.e., gnaw-time and neutrophil count). All statistical analyses was performed using Prism (version 7) statistical software (Graphpad Software Inc., La Jolla, CA, USA) with the exception of the three-way ANOVA, which was performed using SPSS Statistics (IBM Corporation). Results were presented as mean ± standard error of the mean.

## Results

### Sex Differences in Oral Cancer Pain in Patients With Oral Cancer

Using the UCSFOCPQ, 35 women diagnosed with oral cancer reported significantly greater cumulative pain score (median = 287) compared to 37 men with oral cancer (median = 226; *U* = 447.5, *P* = 0.024, Mann-Whitney *U*). Specifically, women with oral cancer reported significantly greater spontaneous and functional pain intensity (Q1: *U* = 453, *P* = 0.028; Q2: *U* = 458, *P* = 0.032), greater sharp/stabbing pain during function (Q4: *U* = 441, *P* = 0.019), and greater functional restriction (Q8: *U* = 433.5, *P* = 0.015) compared to men with oral cancer (Figure 1).

### Sex Differences in Nociceptive Behavior During 4NQO-Induced Carcinogenesis

To establish an oral SCC model that enabled assessment of nociceptive behavior induced by cancer in an intact mouse, C57BL/6 mice ingested the carcinogen, 4NQO, in the drinking water on an unrestricted basis for 16 weeks followed by a 12-week monitoring period (Figure 2A). Control mice received water containing the diluent, propylene glycol (vehicle). Nociceptive behavior was measured using the dolognawmeter assay in which gnaw-time is a validated index of orofacial nociception (Dolan et al., 2010). All 40 female and 40 male mice treated with 4NQO in the drinking water exhibited clinical and pathologic changes in the tongue by 8 weeks after the termination of 4NQO (week 28, Figure 2A). Ten female and 10 male mice treated with propylene glycol (vehicle) showed no clinical or pathologic changes in the oral cavity (Figure 2Ba). Moderate or severe dysplastic changes were detected in 62.3% of female mice and 60.0% of male mice. Oral SCC, both papillary (Figure 2Bb) and invasive (Figure 2Bc), were identified in 37.5% of female mice and 40.0% of male mice. There was no difference in tumor location identified between male and female mice with 4NQO-induced oral SCC (Figure 2C). By contrast, a sex difference in orofacial nociceptive behavior was observed during 4NQO-induced carcinogenesis (Treatment × Sex × Time, *P* = 0.040 Three-way ANOVA; Figure 2D). When comparing males to females, 15 female mice with 4NQO-induced oral SCC exhibited significantly longer gnaw-time compared to 16 males with 4NQO-induced oral SCC at 25–28 weeks (Figure 2D). Additionally, females with 4NQO-induced oral SCC had significantly longer gnaw-time compared to 10 vehicle-treated females at 22–23 weeks (*P* < 0.05) and 24–28 weeks (*P* < 0.01, Figure 2D), whereas males with 4NQO-induced oral SCC had significantly longer gnaw-time compared to 10 vehicle-treated males at 28 weeks only (*P* = 0.002, Figure 2D).

### No Sex Difference in Tongue Primary Afferent Excitability

**Figure 3:**
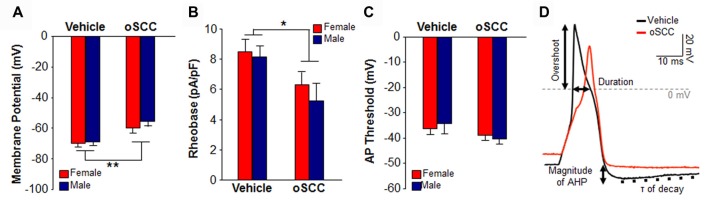
Neuronal sensitization during the progression of 4NQO-induced oral carcinogenesis. Excitability was quantified with resting membrane potential **(A)**, rheobase **(B)** and action potential (AP) threshold **(C)** in trigeminal ganglia (TG) neurons from vehicle-treated mice and 4NQO-treated female (red bars, *N* = 4) and male (blue bars, *N* = 6) mice with oSCC. *P* > 0.05 for Sex vs. Treatment by Two-way ANOVA. Pooled neurons from female and male mice with 4NQO-induced oSCC had significantly depolarized membrane potential and significantly decreased rheobase. *P < 0.05, **P < 0.01 vs. vehicle-treated by Unpaired Student’s *t*-test. **(D)** A representative plot of depolarization (4 ms) evoked AP from a vehicle-treated (black) female mouse and a female mouse with 4NQO-induced oral oSCC (red) showing how we defined the active electrophysiological properties analyzed in Table 1. AHP is afterhyperpolarization; τ is time constant of AHP decay.

Baseline neuronal excitability was not significantly different in retrograde labeled (DiI+) tongue primary afferent neurons from six male mice and four female mice with 4NQO-induced oral SCC (Sex × Treatment, *P* = 0.595 for membrane potential, *P* = 0.571 for Rheobase, *P* = 0.172 for AP threshold, Two-way ANOVA). No difference in the size distribution of acutely dissociated, DiI+ neurons from vehicle-treated mice or mice with 4NQO-induced oral SCC was observed (Table 1). However, when pooled, 23 DiI+ neurons from female and male mice with 4NQO-induced oral SCC exhibited a significantly more depolarized resting membrane potential (−16.3 ± 3.1%, *P* = 0.0002 Unpaired Student’s *t*-test; Figure 3A) and a significantly lower rheobase (−25.6 ± 1.1%, *P* = 0.041; Figure 3B) compared to 31 neurons from vehicle-treated female and male mice. We found no significant difference in AP threshold (11.5 ± 1.7%, *P* = 0.401 Unpaired Student’s *t*-test; Figure 3C). There was also no sex difference in the active electrophysiological parameters, as defined in Figure 3D, in neurons from female and male mice with 4NQO-induced oral SCC (Table 1). When neurons from female and male mice were pooled, the peak amplitude of the AP (overshoot) was significantly smaller (*P* = 0.0001, Unpaired Student’s *t-test*) in 23 neurons from mice with 4NQO-induced oral SCC (45.3 ± 3.4 mV) compared to 31 neurons from vehicle-treated mice (61.7 ± 1.7 mV). While there was no change in the AHP magnitude between groups (*P* = 0.304), the AHP time constant (τ) of decay was significantly increased (*P* = 0.0035, Unpaired Student’s *t*-test) in neurons from mice with 4NQO-induced oral SCC (164.5 ± 15 ms) compared to neurons from vehicle-treated mice (64.5 ± 10 ms).

**Table 1:** Passive and active electrophysiological parameters of the evoked action potential (AP) in tongue afferents from female and male cancer free (vehicle) and 4-nitroquinoline-1-oxide (4NQO)-induced cancer bearing (oral squamous cell carcinoma, oSCC) mice.

	Capacitance (pf)	Input resistance (mΩ)	Overshoot (mV)	Duration (ms)	Mag of AHP (mV)	τ of decay (ms)
**Female**						
Vehicle (*n* = 10)	12.2 ± 1.1	1541.1 ± 224.8	60.7 ± 2.5	3.2 ± 0.3	−15.5 ± 1.5	65.1 ± 12.1
oSCC (*n* = 11)	14.2 ± 1.5	1389.3 ± 453.6	38.3 ± 4.2	2.6 ± 0.2	−16.7 ± 2.1	160.3 ± 33.5
**Male**						
Vehicle (*n* = 13)	13.0 ± 0.7	1763.4 ± 353.6	57.2 ± 1.3	3.4 ± 0.1	−15.9 ± 1.0	63.9 ± 15.6
oSCC (*n* = 12)	15.5 ± 1.1	1611.7 ± 240.5	32.3 ± 4.1	4.2 ± 0.6	−18.2 ± 2.0	168.6 ± 52.1
**Pooled male and female**						
Vehicle (*n* = 23)	14.9 ± 1.0	1686 ± 238.9	61.7 ± 1.7	3.3 ± 0.3	−15.7 ± 0.8	64.5 ± 10
oSCC (*n* = 23)	14.9 ± 0.9	1512 ± 237.5	45.3 ± 3.4**	3.4 ± 0.4	−17.5 ± 1.4	164.5 ± 15**

AHP is afterhyperpolarization, τ is the time constant of AHP decay, Two-way ANOVA, Treatment × Sex interaction P > 0.05; Pooled Male and Female, Unpaired Student’s t-test **P < 0.01.

### Opioid-Mediated Analgesic Mechanism in Male Mice Only

**Figure 4:**
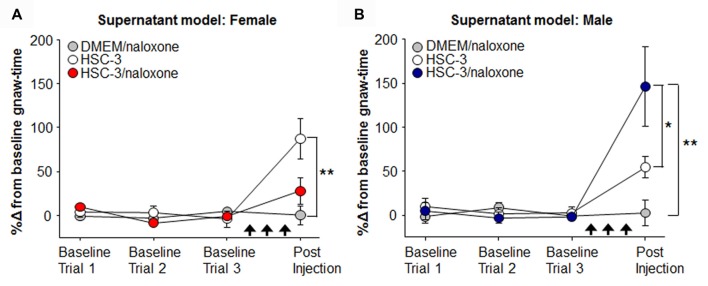
Naloxone potentiation of oral cancer-evoked orofacial pain in male mice. Using the acute oral cancer pain model, female** (A)** and male **(B)** mice received three consecutive injections (black arrows) of either opioid receptor antagonist, naloxone (500 μg/kg) in culture media (DMEM/naloxone, gray circle, *N* = 5 female, 5 male), HSC-3 cell culture supernatant (HSC-3, white circle, *N* = 5 female, 5 male), or HSC-3 cell culture supernatant with naloxone (HSC-3/naloxone, red circles, *N* = 8 **(A)**, blue circles, *N* = 10 **(B)**) followed by assessment in the dolognawmeter (Post Injection Trial). Data were analyzed as a percent change from the baseline gnaw-time, i.e., the mean of the last three dolognawmeter training trials for each animal. *P* < 0.05 for Sex vs. Treatment by Two-way ANOVA, *P < 0.05, **P < 0.01 by Holm-Sidak *post hoc* comparisons.

To measure oral cancer pain behavior in the absence of tumor burden and illness that accompany carcinogenesis, we used the acute oral cancer pain mouse model (Scheff et al., 2017). Using the dolognawmeter assay, baseline gnaw-time was established and mice received three consecutive injections of cell culture supernatant. Nociceptive behavior was measured 1 h after the third injection. Peripheral opioid receptor antagonist, naloxone methiodide, revealed an endogenous analgesic mechanism in male mice. We found a significant interaction between sex and treatment with HSC-3 cell culture supernatant, naloxone in cell culture media (DMEM/naloxone), or naloxone co-injected with HSC-3 supernatant (*P* = 0.006, Two-way ANOVA). Five female mice had significantly longer gnaw-time in response to HSC-3 supernatant (*P* = 0.012) when compared to 5 female mice injected with DMEM/naloxone (Figure 4A). However, when naloxone was co-injected with HSC-3 supernatant, 10 male mice showed significantly longer gnaw-time compared to five males injected with DMEM/naloxone (461.4 ± 70.9%, *P* = 0.001) or five male mice that received HSC-3 alone (7370.5.4 ± 70.7%, *P* = 0.033; Figure 4B). Co-injection with naloxone and HSC-3 supernatant was not significantly different from DMEM/naloxone in eight female mice (*P* = 0.717).

### Sex Difference in Neutrophil Recruitment Associated With 4NQO-Induced Oral SCC

**Figure 5:**
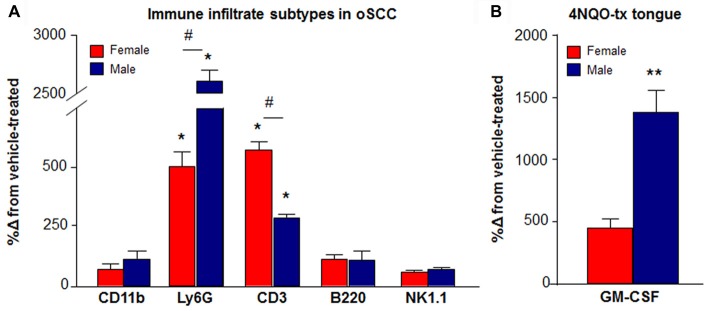
Increased immune cell infiltrate during the development of 4NQO-induced oSCC. **(A)** Using flow cytometry, infiltrating immune cell subpopulations were quantified in dissociated tongue tissue from female (red bars) and male (blue bars) vehicle-treated mice (*N* = 9 females, 9 males) and mice with 4NQO-induced oSCC (*N* = 8 female, 10 males). Data are presented as a percent change from vehicle-treated mice. The percent of total live cells quantified for both vehicle and 4NQO-treated mice with oral SCC are available in Table 2. *P* < 0.05 for Sex vs. Treatment by Two-way ANOVA; *P < 0.05, **P < 0.01 for Holm-Sidak *post hoc* comparisons for treatment; ^#^*P* < 0.05 for Holm-Sidak *post hoc* comparisons for sex. **(B)** Granulocyte macrophage-colony stimulating factor (GM-CSF) protein was measured in homogenized tongue tissue from male (*N* = 5) and female (*N* = 4) mice with 4NQO-induced oSCC compared to vehicle-treated male (*N* = 4) and female (*N* = 4) mice. GM-CSF protein concentration was significantly higher in male mice (blue bar) with 4NQO-induced oSCC compared to female mice (red bar). Data are represented as percent change from vehicle-treated mice. **P < 0.01 by Unpaired Student’s *t*-test.

Sex differences in myeloid and lymphocytic immune cell subpopulations were found in immune infiltrate (CD45^+^ tongue cells) from mice with 4NQO-induced oral SCC compared to vehicle-treated mice. There was a significant interaction between sex and 4NQO treatment for Ly6G^+^ neutrophils (*P* = 0.048) and CD3^+^ T cells (*P* = 0.048, Two-way ANOVA). Significantly more Ly6G^+^ neutrophils were quantified in tongues from 10 male mice with 4NQO-induced oral SCC compared to tongues from nine vehicle-treated male mice (2605.8 ± 97.7%, *P* = 0.002) and eight female mice with 4NQO-induced oral SCC (121.1 ± 10.3%, *P* = 0.026). Alternatively, significantly more CD3^+^ T cells were quantified in eight female mice with 4NQO-induced oral SCC compared to nine vehicle-treated female mice (980.5 ± 33.6%, *P* < 0.0001) and 10 male mice with 4NQO-induced oral SCC (65.6 ± 3.5%, *P* = 0.046; Figure 5A). There was no significant interaction between sex and 4NQO treatment in the number of monocytes/macrophages (*P* = 0.632), B cells (*P* = 0.800), or NK cells (*P* = 0.768) quantified (Two-way ANOVA, Figure 5A). Data expressed as a percent of live cells quantified from both 4NQO and vehicle treatment are available in Table 2.

**Table 2:** Immune cell subpopulations from female and male cancer free (vehicle) and 4NQO-induced cancer bearing (oSCC) mice.

CD45^+^ cells	Female		Male	
	Vehicle	oSCC	Vehicle	oSCC
CD11b^+^	1.48 ± 0.14	2.58 ± 0.17	1.1 ± 0.14	2.41 ± 0.30
Ly6G^+^	0.24 ± 0.05	1.39 ± 0.33*	0.11 ± 0.02	3.07 ± 0.97*,^#^
CD3^+^	0.13 ± 0.03	1.42 ± 0.33*,^#^	0.23 ± 0.01	0.86 ± 0.12*
B220^+^	0.31 ± 0.08	0.65 ± 0.15	0.43 ± 0.09	0.90 ± 0.37
NK1.1^+^	0.12 ± 0.03	0.19 ± 0.03	0.13 ± 0.04	0.22 ± 0.06

Data is presented as percent of total live cells counted, Two-way ANOVA, Treatment × Sex interaction P < 0.05, Holm-Sidak post hoc analysis for Treatment, *P < 0.05, for Sex ^#^P < 0.05.

A sex difference in the tongue tissue of mice with 4NQO-induced oral SCC compared to vehicle-treated mice was also found in the protein concentration of granulocyte macrophage-colony stimulating factor (GM-CSF), a prominent cytokine secreted at high concentration by oral cancer (Scheff et al., 2017) and responsible for circulating neutrophil recruitment (Shi et al., 2006). Significantly more GM-CSF protein was present in five tongues from male mice with 4NQO-induced oral SCC compared to four tongues from female mice with 4NQO-induced oral SCC (243.2 ± 9.3%, *P* = 0.015 Unpaired Student’s *t*-test; Figure 5B).

### Leukocytes in the Oral Cancer Microenvironment Express Opioids

**Figure 6:**
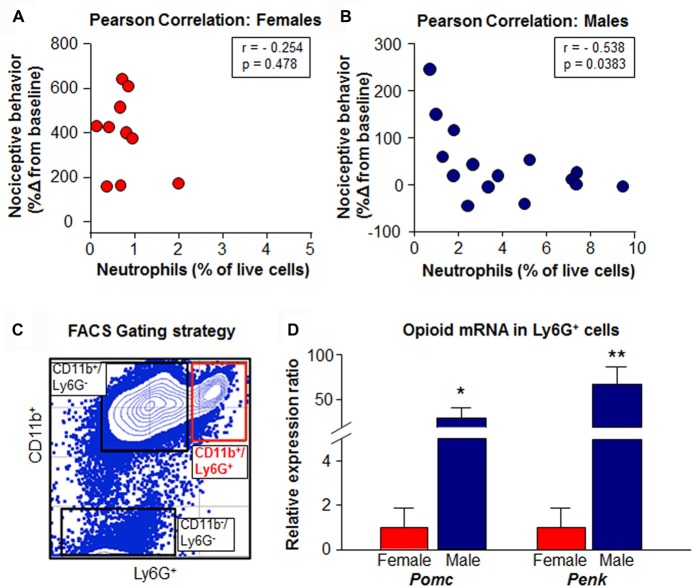
Opioid expression in cancer-stimulated immune cell subpopulations. The relationship between the number of Ly6G^+^ neutrophils in the tongue oSCC microenvironment and measured nociceptive behavior expressed as a percent change from baseline in **(A)** female (*N* = 10, *P* > 0.05) and **(B)** male (*N* = 15, *P* < 0.05) mice was evaluated with Pearson correlation. Note nociceptive behavior data in **(A,B)** are plotted on different scales due a greater spread in females. Following three consecutive injections of HSC-3 supernatant, mouse tongue tissue was dissociated and immune cell subpopulations were separated and collected for analysis by quantitative real-time polymerase chain reaction (qRT-PCR). **(C)** Representative gating strategy used to isolate cancer-activated tongue immune cells by fluorescence-activated cell sorting (FACS). **(D)** Quantification of mean *Pomc* and *Penk* expression in CD11b^+^Ly6G^+^ and CD11b^+^Ly6G^−^ immune cell subpopulations from HSC-3 supernatant-treated male (blue, *N* = 4) and female mice (red, *N* = 4) relative to housekeeping gene *Actb*. Data were analyzed using the Pfaffl method and normalized to the average expression ratios from female mice. *P < 0.05, **P < 0.01 by Unpaired Student’s *t*-test.

Neutrophil infiltration negatively correlated with nociceptive behavior in 15 male mice (*r* = −0.538, *P* = 0.038) but not in 10 female mice (*r* = −0.254, *P* = 0.478, Pearson correlation; Figures 6A,B). To determine if cancer-recruited neutrophils express opioid precursor mRNA, CD45^+^CD11b^+^Ly6G^+^ cells were isolated after HSC-3 supernatant treatment from mouse tongues using FACS (Figure 6C). Presence of *Pomc* and *Penk* transcripts was detected in cancer supernatant-recruited neutrophils isolated from four male and Four female mice, but prodynorphin (*Pdyn*) was below the level of detection. Furthermore, *Pomc* and *Penk* mRNA expression in neutrophils isolated from male mice was significantly greater than in neutrophils isolated from female mice (*P* = 0.031 for *Pomc* and *P* = 0.006 for *Penk*, Unpaired Student’s *t*-test; Figure 6D).

### Monoclonal Antibodies Against Ly6G^+^ Can Deplete Neutrophils in the Tongue Microenvironment

**Figure 7:**
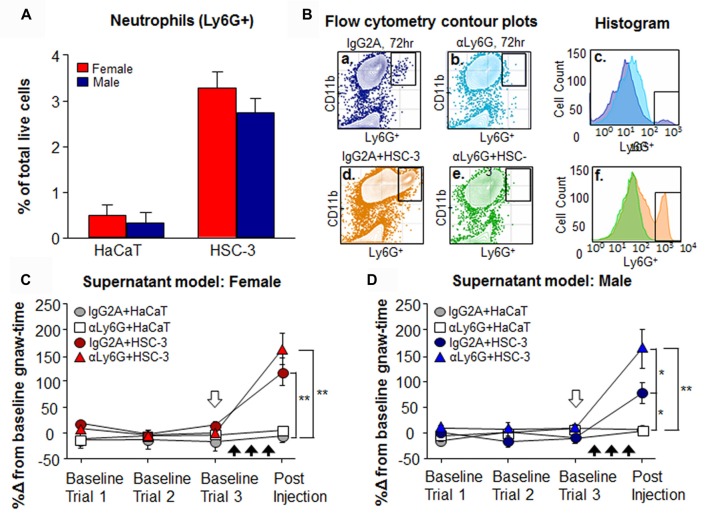
Monoclonal antibody (mAb) depletion of Ly6G^+^ neutrophils in the tongue microenvironment.** (A)** Using flow cytometry, average Ly6G^+^ neutrophils were quantified in tongue tissue from female (*N* = 10) and male mice (*N* = 12) following three consecutive injections of HSC-3 or HaCaT cell culture supernatant. *P* > 0.05 by Two-way ANOVA. **(B)** Representative scatter plots showing CD11b^+^Ly6G^+^ neutrophil gating in a naïve male mouse 72 h after treatment with **(a)** IgG2A isotype control (dark blue) or depletion of neutrophils by **(b)** αLy6G (light blue). **(c)** Histogram demonstrates the loss of Ly6G^+^ neutrophils by αLy6G (light blue) in a naïve male mouse. **(d)** IgG2A isotype control treatment (orange) and **(e)** αLy6G-mediated depletion (green) 12 h prior to the acute supernatant model with HSC-3 cell culture supernatant. **(f)** Histograms demonstrate the absence of HSC-3-induced neutrophil infiltration after αLy6G (green). **(C,D)** Neutrophil depletion mediated change in nociceptive behavior was examined using the acute supernatant model. After establishment of baseline gnaw-time, mice were administered a single i.p. injection of αLy6G or IgG2A isotype control (white arrow). After 12 h. HSC-3 or HaCaT supernatant was injected into the tongue for three consecutive days (black arrows) and supernatant-induced change in nociceptive behavior was measured in **(C)** female and **(D)** male mice. *N* = 10/group. *P < 0.05, **P < 0.01 by One-way ANOVA, Holm-Sidak *post hoc*. There was no significant interaction between sex and treatment. *P* > 0.05 by Two-way ANOVA.

HSC-3 supernatant injection into the tongue resulted in an increase in Ly6G^+^ neutrophils into the cancer microenvironment in 12 male and 10 female mice compared to supernatant from non-tumorigenic keratinocyte cell line, HaCaT (Figure 7A). However, there was no significant interaction between sex and treatment (*P* = 0.218, Two-way ANOVA). Systemic treatment of the anti-mouse Ly6G mAb (αLy6G) in a healthy mouse resulted in full depletion of Ly6G^+^ cells in the tongue by 12 h and lasted at least 72 h (Figures 7Ba–c). Similarly, αLy6G treatment prevented neutrophil recruitment in response to three consecutive HSC-3 supernatant injections (Figures 7Bd–f). To determine the effect of neutrophil infiltration on acute oral cancer pain, we depleted neutrophils in the acute oral cancer pain model. Female and male mice were administered a single i.p. injection of either αLy6G or IgG2A isotype control. After 12 h, HSC-3 or HaCaT cell culture supernatant was injected into the tongue for three consecutive days. There was no significant interaction between sex and treatment (*P* = 0.262, Two-way ANOVA). However, analysis of each sex independently found that HSC-3 cell culture supernatant in the presence of either IgG2A isotype control or αLy6G resulted in significantly longer gnaw-time compared to HaCaT supernatant in both female (*P* = 0.0001 for IgG2A, *P* < 0.0001 for αLy6G, One-way ANOVA, Figure 7C) and male mice (*P* = 0.039 for IgG2A and *P* = 0.0002 for αLy6G, One-way ANOVA; Figure 7D). However, in male mice, HSC-3 supernatant injection in the presence of αLy6G resulted in significantly longer gnaw-time compared to HSC-3 supernatant in the presence of IgG2A isotype control (*P* = 0.020, One-way ANOVA; Figure 7D).

### Loss of Neutrophils During Carcinogenesis Results in Nociceptive Behavior in Males

**Figure 8:**
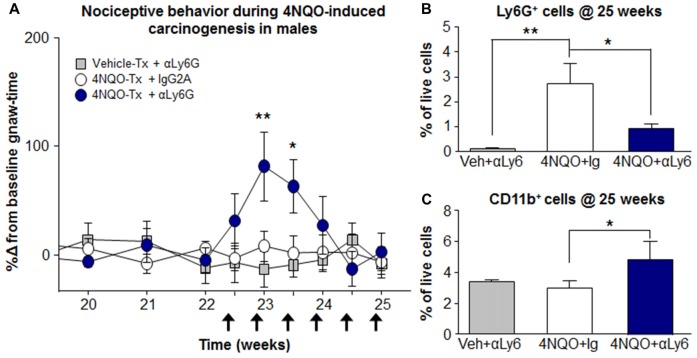
Potentiation of nociceptive behavior in male mice secondary to loss of neutrophils during carcinogenesis.** (A)** 4NQO carcinogenesis-induced change in nociceptive behavior was measured in male mice receiving IgG2A (*N* = 9, white circles) or αLy6G (*N* = 10, blue circles) every 72 h (black arrows) compared to vehicle-treated male mice receiving αLy6G (*N* = 5, gray squares). **P < 0.01 for treatment vs. time by Two-way ANOVA. *P < 0.05, **P < 0.01 by Holm-Sidak *post hoc*. **(B,C)** Comparison of immune cell infiltration into the tongue measured by flow cytometry at week 25 during 4NQO carcinogenesis. Mice received IgG2A (white bars, 4NQO+IgG, *N* = 9) or αLy6G (blue bars, 4NQO + Ly6, *N* = 10). Infiltrate is compared to vehicle-treated mice receiving αLy6G (gray bars, Veh + Ly6, *N* = 5). *P < 0.05, **P < 0.01 by One-way ANOVA, Holm-Sidak *post hoc*.

In the presence of 4NQO-induced oral SCC, an abundance of neutrophils were evident in the oral cancer microenvironment in male mice (Figure 5A). We subsequently identified an analgesic role for neutrophils in male mice during 4NQO-induced carcinogenesis using chronic antibody-mediated neutrophil depletion by repeated αLy6G injection (Treatment by time interaction *P* = 0.003, Two-way ANOVA). There was a significant increase in gnaw-time in αLy6G-treated mice compared to IgG2A isotype control at week 23 (*P* = 0.004) and week 23.5 (*P* = 0.021) in the 4NQO oral cancer model (Figure 8A). However, the increased nociceptive behavior was not sustained for the duration of αLy6G treatment; nociceptive behavior returned to baseline at week 24 of chronic neutrophil depletion (*P* = 0.710, Figure 8A). At week 25, quantification of immune cells in the tongue revealed significantly less neutrophil recruitment in αLy6G-treated mice compared to IgG2A-treated mice during 4NQO carcinogenesis (−65.16 ± 2.5%, *P* = 0.033, One-way ANOVA; Figure 8B). However, neutrophil recruitment in 4NQO-treated mice receiving αLy6G was still significantly greater than vehicle-treated mice receiving αLy6G (695.7 ± 5.1%, *P* = 0.009, One-way ANOVA; Figure 8B). Furthermore, tongues from 4NQO-treated mice receiving αLy6G also had a significant increase in CD11b^+^Ly6G^−^ monocyte/macrophage infiltration into the cancer microenvironment compared to IgG2A-treated mice (154.9 ± 36.7%, *P* = 0.028, One-way ANOVA; Figure 8C) suggesting a compensatory response to the αLy6G-induced decrease in neutrophil infiltration.

## Discussion

We report a mechanism to explain the sex difference in oral cancer pain. Our clinical and preclinical findings of increased cancer pain in females are consistent with results by Reyes-Gibby et al. (2014) who show that women with oral cancer report more pain than men across 2,340 subjects. Here, we report that tongue afferent neuronal excitability in female and male mice with oral cancer is similar; therefore, primary neuronal plasticity is unlikely the explanation for the sex difference. Although inflammation is a hallmark of cancer (Hanahan and Weinberg, 2011), little is known about the contribution of the cancer-associated immune infiltrate to oral cancer pain. Wang et al. (2014) show that neutrophil infiltration is more abundant in human tongue SCC tissues and that the neutrophil density is higher in men compared to women. Our investigation of inflammation reveals differential neutrophil infiltration in the 4NQO oral cancer microenvironment of female and male mice. However, neutrophil-mediated analgesia inhibits nociceptive behavior only during the early stage of 4NQO-induced oral cancer development. The antinociceptive effect following αLy6G-mediated neutrophil depletion is lost by 24 weeks. Possibly, a reduction in neutrophil recruitment during 4NQO-induced carcinogenesis results in a delayed compensatory increase in CD45^+^CD11b^+^ monocyte/macrophages, or other leukocyte subpopulations, that contribute to nociception (Przewłocki et al., 1992; Plein and Rittner, 2017). The underlying cause of the sex difference in neutrophil infiltration remains unidentified; however, sex hormones are a potential candidate. Estrogen affects the number of circulating neutrophils and neutrophil lifespan (Bouman et al., 2005). Studies using injury and burn rodent models find that testosterone potentiates, whereas estrogen limits Ca^2+^ mobilization in neutrophils (Deitch et al., 2006), suggesting that gonadal hormones can regulate neutrophil activity.

Oral cancer patients consistently report significantly higher function-related pain rather than spontaneous pain (Connelly and Schmidt, 2004). We found a significant sex difference in functional intensity, functional sharpness and functional restriction in our clinical patient cohort. In an attempt to recapitulate these clinical findings in rodents, we measured a behavioral index of gnawing-induced nociception with the dolognawmeter assay. Gnawing is a routine orofacial function that is coordinated by the trigeminal somatosensory and motor systems and activates the temporomandibular joint, muscles of mastication, jaws, incisors, lips, tongue, buccal mucosa, palate and gingiva in a fashion that is similar to the chewing associated with mastication in humans. In addition to function-related pain, we also found a significant sex difference in reported intensity of spontaneous pain. Conditioned place preference could be used to test the hypothesis that there is a sex difference in spontaneous pain secondary to oral cancer in mice (King et al., 2009).

We find that cancer-recruited neutrophils express β-endorphin and met-enkephalin, which are antinociceptive in male mice; neutrophil depletion produces nociception. Immune cells drive endogenous antinociception in non-cancer pain. Opioid-containing leukocytes contribute to endogenous pain inhibition during early complete Freund’s adjuvant (CFA)-induced inflammatory pain (Mousa et al., 2004) and in models of chronic neuropathic pain (Labuz et al., 2009; Chao et al., 2012). Labuz et al. (2009) report that immune infiltration during nerve injury produces suppression of mechanical allodynia by the secretion of opioids. Similar to our results with naloxone, immune-mediated anti-allodynia is blocked by naloxone methiodide. Exogenous granulocyte-colony stimulating factor alleviates thermal hyperalgesia and mechanical allodynia in rats with chronic constriction injury through leukocyte-derived endogenous opioids (Chao et al., 2012). In humans, opioid peptides released locally by leukocytes decrease pain intensity following surgery (Stein et al., 2003). Awad et al. (2012) report that neutrophils in a wound, collected from sternotomy patients, contain high levels of endogenous opioids and possibly contribute to peripheral analgesia.

Animal and human data support the role of peripheral opioids for analgesia (Stein et al., 1990; Kapitzke et al., 2005). Administration of the peripherally restricted opioid morphine-6-glucoronide reduces hyperalgesia induced by freezing skin to −30°C and delayed onset muscle soreness (Tegeder et al., 2003). Opioid peptides from keratinocytes contribute to endogenous analgesia following administration of endothelin-1 (Viet et al., 2011). Oral carcinoma is comprised of malignant oral keratinocytes, which might serve as a potential source of opioids within the cancer microenvironment. Viral- and nonviral-mediated delivery of genes for the μ-opioid receptor and the endothelin B receptor (*OPRM1* and *EDNRB*, respectively) produce endogenous analgesia through the secretion of opioids by the carcinoma in preclinical oral cancer pain models (Viet et al., 2011; Yamano et al., 2017). Adenoviral transduction produces immune effects that prohibit clinical use (Nayak and Herzog, 2010). Furthermore, concerns for adenoviral-mediated transduction of cancer include limited transduction efficiency and replication specificity (Yamamoto and Curiel, 2010). However, there are no studies in cancer pain for immune-mediated endogenous analgesia. Analgesia targeted to the cancer microenvironment obviates off-target effects of opioids in the CNS and gastrointestinal tract. Immune-mediated endogenous analgesia could be enhanced through immune cell number (Chao et al., 2012) or opioid peptide production (Chuang et al., 2005). Neutrophils, T cells and macrophages contain opioid peptide mRNA (Przewłocki et al., 1992; Plein and Rittner, 2017). Recently developed immunotherapy for cancer (Ferris et al., 2016) may increase infiltration of opioid-containing immune cells that could inhibit pain as well as reduce tumor burden.

There are three limitations of our experimental design. The first is the lack of comprehensive neutrophil characterization within the cancer microenvironment. Neutrophils in mice are recognized as CD11b^+^Ly6G^+^ (Daley et al., 2008; Fridlender and Albelda, 2012), markers which do not differentiate between neutrophil subtypes. The cancer microenvironment can be infiltrated by anti- (N1) or pro-tumoral (N2) neutrophils (Fridlender and Albelda, 2012; Sagiv et al., 2015). N1 and N2 neutrophil phenotypes are morphologically similar; however, transcriptional profiling can distinguish them (Elpek et al., 2014). Single-cell analysis might allow for classification of N1 and N2 infiltrating neutrophils during 4NQO-induced carcinogenesis in the mouse model. The second limitation is the inconsistent outcomes in the two cancer models with sex differences and neutrophil recruitment. While we find a sex difference in neutrophil infiltration with the 4NQO model there is not a difference in the HSC-3 supernatant-induced model. The administration of oral cancer cell culture supernatant allows for us to isolate the nociceptive effect of inflammatory cell infiltration in the absence of the cancer. However, the supernatant model lacks cancer microenvironment constituents (e.g., tumor cells, cancer-associated fibroblasts) that play a role in immune cell recruitment (Le Bitoux and Stamenkovic, 2008). A final limitation is that we did not monitor gonadal hormones. Sex differences in endogenous analgesia depend on hormonal regulation. Female rats exhibit less swim stress-induced analgesia (SIA); furthermore, swim SIA is reversed by opioid blockade in males, but not in females (Romero et al., 1987, 1988). Mogil et al. (1993) find that estrogen contributes to sex-dependent efficacy of naloxone during swim SIA. We inferred that a component(s) of the oral cancer microenvironment is the source of opioid-mediated analgesia in male mice based on our result that peripherally restricted naloxone methiodide (Rohde et al., 1997) increased HSC-3 supernatant-induced nociceptive behavior in male mice only. Naloxone had the opposite effect in female mice. The underlying mechanism for the antinociceptive effect of naloxone in female mice is unknown. Additional experiments to address this limitation are to repeat the study using gonadectomized mice or administer gonadal hormones.

Our data highlight sex differences in oral cancer pain, which we attribute to endogenous opioids secreted by neutrophils. Our findings suggests that sex is an important variable when considering treatment for oral cancer patients. Female patients may benefit more from peripherally restricted opioids due to a decreased opioidergic neutrophil presence in the tumor microenvironment. Furthermore, therapeutic approaches to activate the immune response in the cancer microenvironment in both sexes are potentially a strategy for the treatment of oral cancer associated pain. Such an approach would avoid the systemic side effects of opioid-based treatments.

## Author Contributions

All authors listed contributed substantially to the work. NS designed the research, conducted the experiments, performed data analyses and wrote the manuscript. AB provided oral histopathological data analyses and guidance. ED and RD provided technical support and data collection for all behavior experiments. JD provided animal behavior expertise and technical support. SW and HK provided technical support and data analysis for oral cancer patient pain questionnaires. BS and DA assisted in research design and writing of the manuscript.

## Conflict of Interest Statement

The authors declare that the research was conducted in the absence of any commercial or financial relationships that could be construed as a potential conflict of interest.
