# Supplementary material for: Neutrophil-Mediated Endogenous Analgesia Contributes to Sex Differences in Oral Cancer Pain
Source: Front Integr Neurosci. 2018 Oct 22;12:52. doi: 10.3389/fnint.2018.00052 (PMC6204375; doi:10.3389/fnint.2018.00052)
Supplement: Supplementary file 1 [file Image_1.pdf]

ORAL PAIN QUESTIONNAIRE

Mark on the line after each question the level of pain you have experienced during the past week:

1. When you *are* ***not*** talking, eating or drinking how ***intense*** (severe, strong) is the pain in your mouth?

   No pain |——————————| The most intense pain sensation imaginable ☐

2. When you *are* talking, eating or drinking how ***intense*** (severe, strong) is the pain in your mouth?

   No pain |——————————| The most intense pain sensation imaginable ☐

3. When you *are* ***not*** talking, eating or drinking how ***sharp*** (like a knife) is the pain in your mouth?

   No pain |——————————| The most sharp pain sensation imaginable ☐

4. When you *are* talking, eating or drinking how ***sharp*** (like a knife) is the pain in your mouth?

   No pain |——————————| The most sharp pain sensation imaginable ☐

5. When you *are* ***not*** talking, eating or drinking how ***aching*** (like a throbbing muscle ache or toothache) is the pain in your mouth?

   No pain |——————————| The most aching pain sensation imaginable ☐

6. When you *are* talking, eating or drinking how ***aching*** (like a throbbing muscle ache or toothache) is the pain in your mouth?

   No pain |——————————| The most aching pain sensation imaginable ☐

7. How ***sensitive*** (like sunburned or raw skin) is the area in your mouth to touch by your teeth, food or fluids?

   No sensitivity |——————————| The most sensitive pain sensation imaginable ☐

8. How significantly does the pain in your mouth restrict your talking, eating or drinking?

   No restriction |——————————| The most severe restriction imaginable ☐

Coordinator's Signature:____ Date: ______________
mm/dd/yyyy

Reprinted from The Journal of Pain, Vol 5/Issue 9, S. Thaddeus Connelly, Brian L. Schmidt, Evaluation of pain in patients with oral squamous cell carcinoma, Page 6, Copyright (2004), with permission from Elsevier.
