# Supplementary material for: Neutrophil-Mediated Endogenous Analgesia Contributes to Sex Differences in Oral Cancer Pain
Source: Front Integr Neurosci. 2018 Oct 22;12:52. doi: 10.3389/fnint.2018.00052 (PMC6204375; doi:10.3389/fnint.2018.00052)
Supplement: Supplementary file 2 [file Table_1.docx]

**Supplemental Table 1:** Clinical characteristics of oral cancer patients

|  | **Number** | **Percent** |
| --- | --- | --- |
| **Age** | | |
| <65  >65 | 33  39 | 46  54 |
| **Sex** | | |
| Women  Men | 35  37 | 49  51 |
| **Site** | | |
| Buccal mucosa  FOM  Gingiva  Retromolar trigone  Tongue | 4  5  22  1  40 | 6  7  31  1  56 |
| **Nodal status** | | |
| N0  N+  Unknown | 35  31  6 | 49  43  8 |
| **T stage** | | |
| T1  T2  T3  T4 | 31  23  4  14 | 43  32  6  19 |
| **Alcohol use** | | |
| Current  Never  Previous  Unknown | 40  13  17  2 | 56  18  24  3 |
| **Tobacco use** | | |
| Current  Never  Previous  Unknown | 16  24  31  1 | 22  33  43  1 |
